# A target-activated AS1411 aptamer probe for *in vivo* and *ex vivo* imaging of nucleolin-overexpressing triple-negative breast cancer

**DOI:** 10.3389/fbioe.2026.1934598

**Published:** 2026-09-23

**Authors:** Juntao Tan, Zixi Hu, Da Huang, Lu Chen, Liangliang Min, Kui Wang, Zhihua Li

**Affiliations:** 1 Jiangxi Province Key Laboratory of Breast Diseases, Nanchang People’s Hospital/The Affiliated Nanchang People’s Hospital of Nanchang Medical College, Nanchang, Jiangxi, China; 2 Clinical Laboratory Center, The First Affiliated Hospital of Guilin Medical University, Guilin, Guangxi, China; 3 Key Laboratory of Tumor Functional Genomics and Precision Prevention and Treatment, Guilin Medical University, Guilin, Guangxi, China; 4 Department of Breast Surgery, Nanchang People’s Hospital/The Affiliated Nanchang People’s Hospital of Nanchang Medical College, Nanchang, China

**Keywords:** activatable probe, AS1411, fluorescence imaging, molecular imaging, nucleolin, triple-negative breast cancer

## Abstract

**Background:**

Fluorescence imaging of breast cancer is often limited by high background signals from conventional “always-on” probes. The nucleolin-targeting AS1411 aptamer provides a promising recognition element for tumors with elevated nucleolin expression, including triple-negative breast cancer (TNBC). This study aimed to develop a target-activated molecular probe to enable high-contrast detection of nucleolin-overexpressing breast tumors.

**Methods:**

An activatable probe (AAFP-AS1411) based on the AS1411 aptamer was engineered by hybridizing fluorescent AS1411 with a quencher strand to generate a target-responsive “OFF-to-ON” fluorescence response. Its preferential fluorescence response and concentration-dependent behavior were evaluated *in vitro* using flow cytometry, fluorescence spectroscopy, and confocal microscopy. *In vivo* tumor-associated fluorescence, *ex vivo* tissue fluorescence distribution, and preliminary acute safety were evaluated in MDA-MB-231 tumor-bearing mice.

**Results:**

AAFP-AS1411 exhibited low background interference and preferential fluorescence activation in MDA-MB-231 TNBC cells compared with MCF-10A cells, with reduced signals following sequence mismatch or competitive blocking. *In vivo*, preferential tumor-associated fluorescence was observed, with the strongest signal detected at 6 h post-injection. *Ex vivo* analysis showed higher fluorescence in tumor tissue than in the examined major organs. No evident acute toxicity was observed at the tested dose of 2 nmol per mouse and the 24-h time point based on hematological, biochemical, and histopathological assessments.

**Conclusion:**

AAFP-AS1411 enabled preferential, high-contrast fluorescence imaging in the tested MDA-MB-231 TNBC model across *in vitro*, *in vivo*, and *ex vivo* experiments. These findings provide proof-of-concept support for further evaluation of activatable AS1411-based probes for molecular imaging of nucleolin-overexpressing TNBC.

## Introduction

Breast cancer remains one of the most common malignancies worldwide and comprises multiple molecular subtypes requiring individualized diagnostic and therapeutic strategies (Sung et al., 2021; Agnello et al., 2023). Among its molecular subtypes, triple-negative breast cancer (TNBC)—characterized by the absence of estrogen receptor (ER) and progesterone receptor (PR) expression and the lack of HER2 overexpression or amplification—is generally associated with aggressive biological behavior and limited availability of subtype-specific therapeutic targets (Jie et al., 2025; Sitia et al., 2022). TNBC accounts for approximately 10%–20% of breast cancers and has a relatively high risk of early recurrence and distant metastasis (Yang et al., 2023; Xiong et al., 2025). Accordingly, molecular imaging strategies that improve tumor-specific visualization may provide additional value for the detection and characterization of TNBC.

Fluorescence imaging offers high sensitivity and rapid signal acquisition and has been increasingly investigated for cancer detection (Zhao et al., 2024). However, conventional “always-on” fluorescent probes continuously emit fluorescence regardless of target engagement, which may increase background signals and reduce tumor-to-background contrast in complex biological environments (Tian et al., 2024). Activatable probes address this limitation by coupling molecular recognition with fluorescence recovery, thereby preferentially generating signals after target interaction.

Aptamers are short single-stranded DNA or RNA molecules that fold into defined three-dimensional structures and recognize molecular targets with high affinity and specificity (Tan et al., 2025; He et al., 2023; Xiao et al., 2024; Fei et al., 2025). AS1411 is a guanine-rich DNA aptamer that interacts with nucleolin, a multifunctional protein that is aberrantly expressed and redistributed to the cell surface in multiple malignant cell types (Verma et al., 2025; Fabre et al., 2022). Consequently, AS1411 has been widely investigated as a targeting ligand for cancer imaging and targeted delivery. Previous studies have demonstrated activatable aptamer strategies for cancer-cell detection (Lai et al., 2017; Wu et al., 2022). In particular, Zhang et al. developed a nucleolin-activated polyvalent AS1411-based aptamer nanoprobe for the detection of cancer cells through recognition of cell-surface nucleolin (Zhang et al., 2023). However, these studies have mainly focused on cellular detection or relatively complex multivalent/amplification systems, and the feasibility of translating a comparatively simple activatable AS1411 probe to serial time-course *in vivo* fluorescence imaging of breast tumors remains insufficiently explored.

To address this remaining gap, we developed an activatable AS1411-based fluorescence probe (AAFP-AS1411) for imaging nucleolin-overexpressing breast cancer. As illustrated in Figure 1, the probe was designed to remain in a low-fluorescence (“OFF”) state in the absence of target interaction, thereby reducing background interference. In this OFF state, hybridization between the fluorophore-labeled AS1411 strand and the complementary quencher strand brings the fluorophore and quencher into close proximity, resulting in fluorescence quenching. Upon interaction with cell-surface nucleolin, a target-associated structural rearrangement is proposed to increase the separation between the fluorophore and quencher, thereby restoring fluorescence (“ON” state). This OFF-to-ON response is intended to improve signal-to-background contrast compared with constitutively fluorescent probes. In this study, we systematically evaluated AAFP-AS1411 through *in vitro* binding assays, cellular imaging, and serial time-course *in vivo* fluorescence imaging at multiple post-injection time points in tumor-bearing mice, together with *ex vivo* tissue fluorescence distribution and tissue imaging, to assess the feasibility of this activatable probe for breast cancer visualization.

**FIGURE 1:**
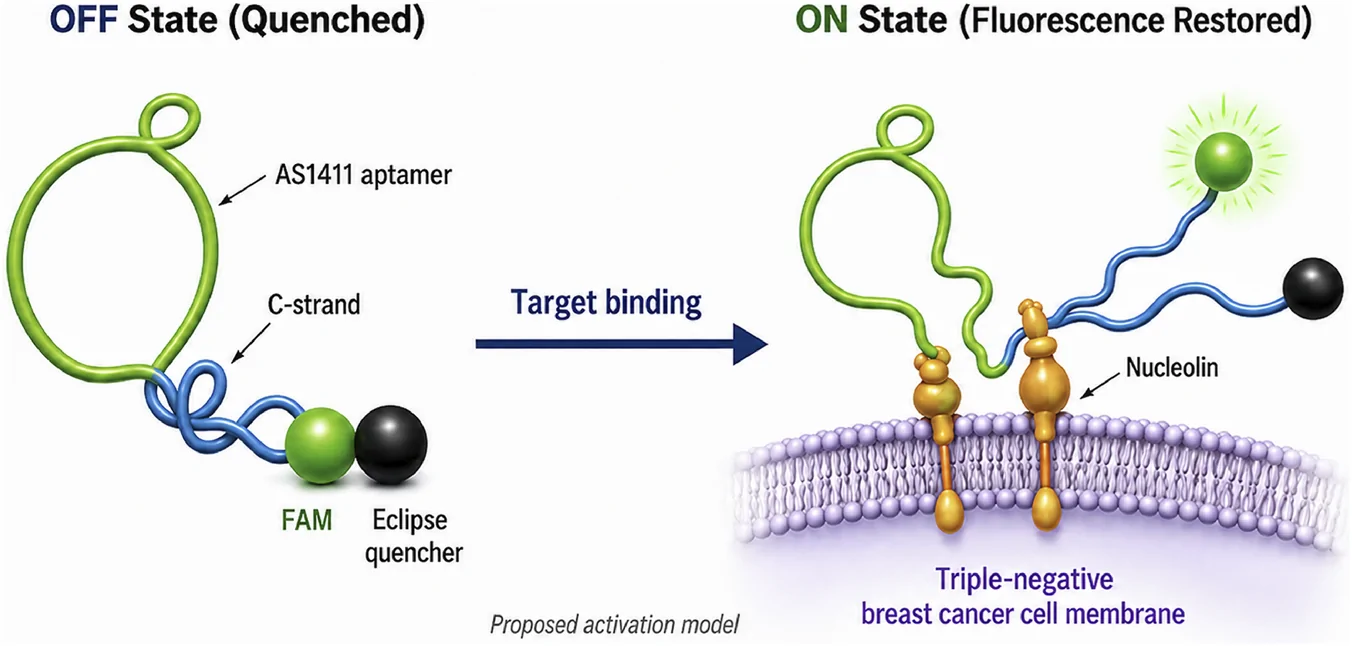
Proposed schematic of the target-responsive AAFP-AS1411 fluorescence probe. AAFP-AS1411 is designed to remain in a quenched low-fluorescence (“OFF”) state through close proximity between the fluorophore and quencher. Upon interaction with nucleolin on the triple-negative breast cancer cell membrane, a target-associated structural rearrangement is proposed to increase fluorophore–quencher separation, resulting in fluorescence recovery (“ON” state). This schematic represents the proposed activation model rather than a directly demonstrated structural mechanism.

## Materials and methods

### Materials

All DNA oligonucleotides were synthesized by Shanghai Sangon Biological Engineering Technology & Services (Shanghai, China). The following oligonucleotides were used in this study: FAM-AS1411: 5′-FAM-GGTTGGTGTGGTTGGTTGGTTGGTTGG-3′ (Fluorescein-labeled AS1411 aptamer); AAFP-AS1411 (AAFP4-AS1411): 5′-FAM-GGGGGGTTGGTGTGGTTGGTTGGTTGGTTGGCCCC-3′ (Activatable fluorescence probe, with four additional C-strand bases); AAFP-AS1411-mis: 5′-FAM-GGGGGGTTGGTGTGGTTGG ***TCGAT*** TGGTTGGTTGGCCCC-3′ (Mismatch control probe, with base substitutions in the C-strand); AAFP3-AS1411: 5′-FAM-GGGGGTTGGTGTGGTTGGTTGGTTGGTTGGCCC-3′(Activatable probe with three additional C-strand bases); AAFP5-AS1411: 5′-FAM-GGGGGGGTTGGTGTGGTTGGTTGGTTGGTTGGCCCCC-3′ (Activatable probe with five additional C-strand bases). The extending C-strand bases are underlined; mismatched bases are shown in bold and italicized. For *in vivo* studies, corresponding probes were synthesized with Cy5.5 at the 5′-end instead of FAM. All oligonucleotides were dissolved in nuclease-free water, quantified by UV-Vis spectroscopy, aliquoted, and stored at −20 °C. RPMI-1640 and DMEM/F12 media, fetal bovine serum (FBS), horse serum, penicillin-streptomycin, L-glutamine, epidermal growth factor (EGF), hydrocortisone, insulin, and cholera toxin were purchased from Gibco (Thermo Fisher Scientific, United States of America). Matrigel was obtained from Corning (United States of America). All other chemicals were of analytical grade.

### Cell culture

The human TNBC cell line MDA-MB-231 and the non-tumorigenic human mammary epithelial cell line MCF-10A were obtained from the American Type Culture Collection (ATCC). MDA-MB-231 cells were cultured in RPMI-1640 medium supplemented with 10% FBS and 1% penicillin-streptomycin. MCF-10A cells were maintained in DMEM/F12 medium supplemented with 5% horse serum, 20 ng/mL EGF, 0.5 μg/mL hydrocortisone, 10 μg/mL insulin, and 100 ng/mL cholera toxin. All cells were maintained at 37 °C in a humidified atmosphere containing 5% CO_2_ and were routinely cultured until approximately 70%–80% confluence, with subculture generally performed every 2–3 days. Cells were routinely tested for *mycoplasma* contamination.

### *In Vitro* experiments

#### Preparation of activatable probes

The activatable probe AAFP-AS1411 was formed by hybridizing the FAM-labeled (or Cy5.5-labeled) AS1411 strand with a complementary quencher strand (IQ-4FQ, integrated DNA Technologies) at a 1:1.2 M ratio in phosphate-buffered saline (PBS, pH 7.4). The mixture was heated to 95 °C for 5 min and then gradually cooled to room temperature over 2 h to facilitate duplex formation, resulting in the quenched “OFF-state” probe. The control probe (AAFP-AS1411-mis) was prepared identically using the mismatched sequence. Probes were used immediately or stored at −20 °C for short periods.

#### Flow cytometry

For binding analysis, cells were harvested, counted, and resuspended in cold PBS containing 1% BSA at a density of 1 × 10^6^ cells/mL. Cell suspensions (100 µL) were incubated with the indicated probes at a final concentration of 250 nM for 30 min at 4 °C in the dark. This low-temperature condition was used as a standardized condition for the initial binding assays to minimize temperature-dependent cellular internalization and trafficking and thereby facilitate assessment of cell-associated probe fluorescence. Temperature-dependent probe responses were separately evaluated at 4 °C, 25 °C, and 37 °C as described below. For competition assays, cells were pre-incubated with a 40-fold molar excess (10 µM) of unlabeled AS1411 for 30 min prior to probe addition. After incubation, cells were washed twice with cold PBS and analyzed immediately using a BD FACSCanto II flow cytometer (BD Biosciences). FAM fluorescence was excited using the 488-nm blue laser and collected using a 530/30-nm bandpass filter. Data from at least 10,000 events per sample were analyzed using FlowJo software (v10.8.1), and mean fluorescence intensity (MFI) was calculated.

#### Fluorescence spectroscopy

Cells were seeded in black-walled 96-well plates at 5 × 10^4^ cells per well and cultured overnight at 37 °C in a humidified atmosphere containing 5% CO_2_ to allow cell attachment. Black-walled plates were used to minimize inter-well optical crosstalk and background fluorescence during signal acquisition. After overnight culture, the plates were removed from the 37 °C incubator, and the cells were washed with PBS to remove the culture medium. Pre-cooled serum-free medium containing 250 nM AAFP-AS1411 was then added, and the cells were incubated at 4 °C for 30 min without an additional temperature-equilibration interval. After incubation, the probe solution was removed and replaced with fresh PBS before spectral acquisition. Fluorescence emission spectra (500–650 nm) were recorded on a Horiba Fluorolog-3 spectrofluorometer with an excitation wavelength of 485 nm (slit widths: 5 nm/5 nm). The intensity at the emission maximum (∼518 nm) was used for quantification. Measurements were performed in triplicate.

#### Confocal laser scanning microscopy (CLSM)

Cells were seeded on glass coverslips in 24-well plates and cultured at 37 °C in a humidified atmosphere containing 5% CO_2_ until approximately 70% confluence. After culture, the cells were removed from the incubator and washed with PBS. Pre-cooled serum-free medium containing 250 nM of the indicated probe was then added, followed by incubation at 4 °C for 30 min without an additional temperature-equilibration interval. After incubation, the cells were washed with PBS and fixed with 4% paraformaldehyde for 15 min. Cell nuclei were counterstained with DAPI (1 μg/mL) for 10 min. After mounting, fluorescence images were acquired using a Zeiss LSM 880 confocal laser scanning microscope equipped with a ×63 oil-immersion objective. FAM and DAPI fluorescence signals were excited using 488-nm and 405-nm laser lines, respectively. Images were processed and analyzed using ZEN software (Zeiss).

### Animal studies

#### Ethics statement

All animal experiments were conducted in strict accordance with the guidelines of the European Convention for the Protection of Vertebrate Animals and were approved by the Animal Ethics Committee of Guilin Medical University (Approval No. GLMU202517013). The work has been reported in accordance with the ARRIVE guidelines (Animals in Research: Reporting *In Vivo* Experiments) (Kilkenny et al., 2010).

#### Tumor Xenograft model

Female BALB/c nude mice (4–5 weeks old, body weight 16–20 g) were used to establish the MDA-MB-231 xenograft model. For tumor cell implantation, mice were anesthetized by inhalation of 1%–2% isoflurane delivered in oxygen at a flow rate of 1 L/min. MDA-MB-231 cells in logarithmic growth phase were harvested, washed, and resuspended in a 1:1 mixture of sterile PBS and Matrigel (Corning). A total of 5 × 10^6^ MDA-MB-231 cells in 100 µL of the suspension were subcutaneously injected into the right flank of each mouse. Tumor growth was monitored twice weekly using a digital caliper, and tumor volume was calculated using the formula: Volume = (length × width^2^)/2. Imaging studies were initiated when tumors reached a volume of approximately 100–150 mm^3^. At the end of the experiments, mice were deeply anesthetized with 5% isoflurane delivered in oxygen at a flow rate of 1 L/min and subsequently euthanized by cervical dislocation.

#### *In vivo and ex vivo* fluorescence imaging

For *in vivo* imaging, MDA-MB-231 tumor-bearing BALB/c mice were randomly divided into three groups (n = 5 per group): (1) AAFP-AS1411 group: injected via the tail vein with 2 nmol of Cy5.5-AAFP-AS1411 in 100 µL of PBS; (2) Mismatch control group: injected with 2 nmol of Cy5.5-AAFP-AS1411-mis; (3) Blocking group: pre-injected with 80 nmol of unlabeled AS1411 30 min prior to administration of 2 nmol of Cy5.5-AAFP-AS1411. Whole-body fluorescence imaging was performed at 1, 6, and 12 h post-injection using an IVIS Spectrum imaging system (PerkinElmer). Acquisition parameters were kept constant (excitation: 675 nm, emission: 720 nm, exposure time: 1 s, binning: medium). Fluorescence intensity within the tumor region of interest (ROI) was quantified using Living Image software (v4.7.3).

Although the strongest tumor-associated fluorescence was observed at 6 h during serial time-course *in vivo* imaging, 12 h was predefined as the terminal time point for tissue collection and *ex vivo* fluorescence analysis to characterize post-peak tissue fluorescence distribution. At 12 h post-injection, mice were euthanized, and the major organs (heart, liver, spleen, lung, and kidneys) and tumors were excised, rinsed with PBS, and imaged *ex vivo* under the same acquisition settings. Fluorescence intensity in each tissue was quantified by ROI analysis to compare the relative fluorescence distribution among the tumor and examined major organs.

#### Histological and *ex vivo* CLSM analysis

Portions of excised tumors and contralateral normal mammary fat pads were embedded in optimal cutting temperature (OCT) compound and snap-frozen. Tissue sections (8 µm thick) were prepared using a cryostat. For probe localization studies, some fresh-frozen tumor and normal tissue sections were incubated with 250 nM Cy5.5-AAFP-AS1411 (or control probes) in PBS for 30 min at 4 °C, washed, fixed, and counterstained with DAPI. All sections were imaged with the Zeiss LSM 880 microscope. Cy5.5 and DAPI signals were captured using 633 nm and 405 nm lasers, respectively.

### Safety evaluation in tumor-bearing mice

To assess the potential acute toxicity of AAFP-AS1411, a separate cohort of MDA-MB-231 tumor-bearing BALB/c nude mice (n = 4 per group) received a single intravenous injection of either PBS (control) or 2 nmol of Cy5.5-AAFP-AS1411 in 100 µL PBS. At 24 h post-injection, blood was collected via retro-orbital bleeding for hematological analysis (white blood cell count (WBC), red blood cell count (RBC), hemoglobin (HGB), and platelet count (PLT)) and assessment of liver/kidney function (aspartate aminotransferase (AST), alanine aminotransferase (ALT), blood urea nitrogen (BUN), and creatinine (CREA)). Subsequently, mice were euthanized, and major organs (heart, lung, liver, spleen, and kidney) were harvested, fixed in 4% paraformaldehyde, paraffin-embedded, sectioned, and stained with hematoxylin and eosin (H&E) for histological examination by a pathologist blinded to the treatment groups.

#### Statistical analysis

All quantitative data are presented as mean ± standard deviation (SD). Statistical analysis was performed using GraphPad Prism software (v9.0). Differences between two groups were analyzed by unpaired two-tailed Student’s t-test. Comparisons among multiple groups were performed using one-way analysis of variance (ANOVA) followed by Tukey’s *post hoc* test for multiple comparisons. A p-value of less than 0.05 was considered statistically significant.

## Results

### Target-associated binding and fluorescence response of AAFP-AS1411

The binding selectivity of AAFP-AS1411 was evaluated in MDA-MB-231 breast cancer cells and non-tumorigenic MCF-10A mammary epithelial cells using flow cytometry and fluorescence spectroscopy. Flow cytometric analysis (Figure 2A) revealed markedly higher fluorescence intensity in MDA-MB-231 cells treated with AAFP-AS1411 than in MCF-10A cells, indicating a preferential fluorescence response in the breast cancer cell model. In contrast, the mismatch control probe (AAFP-AS1411-mis) produced only minimal fluorescence in both cell lines (Figure 2B), supporting the importance of the intact AS1411 sequence for the observed fluorescence response.

**FIGURE 2:**
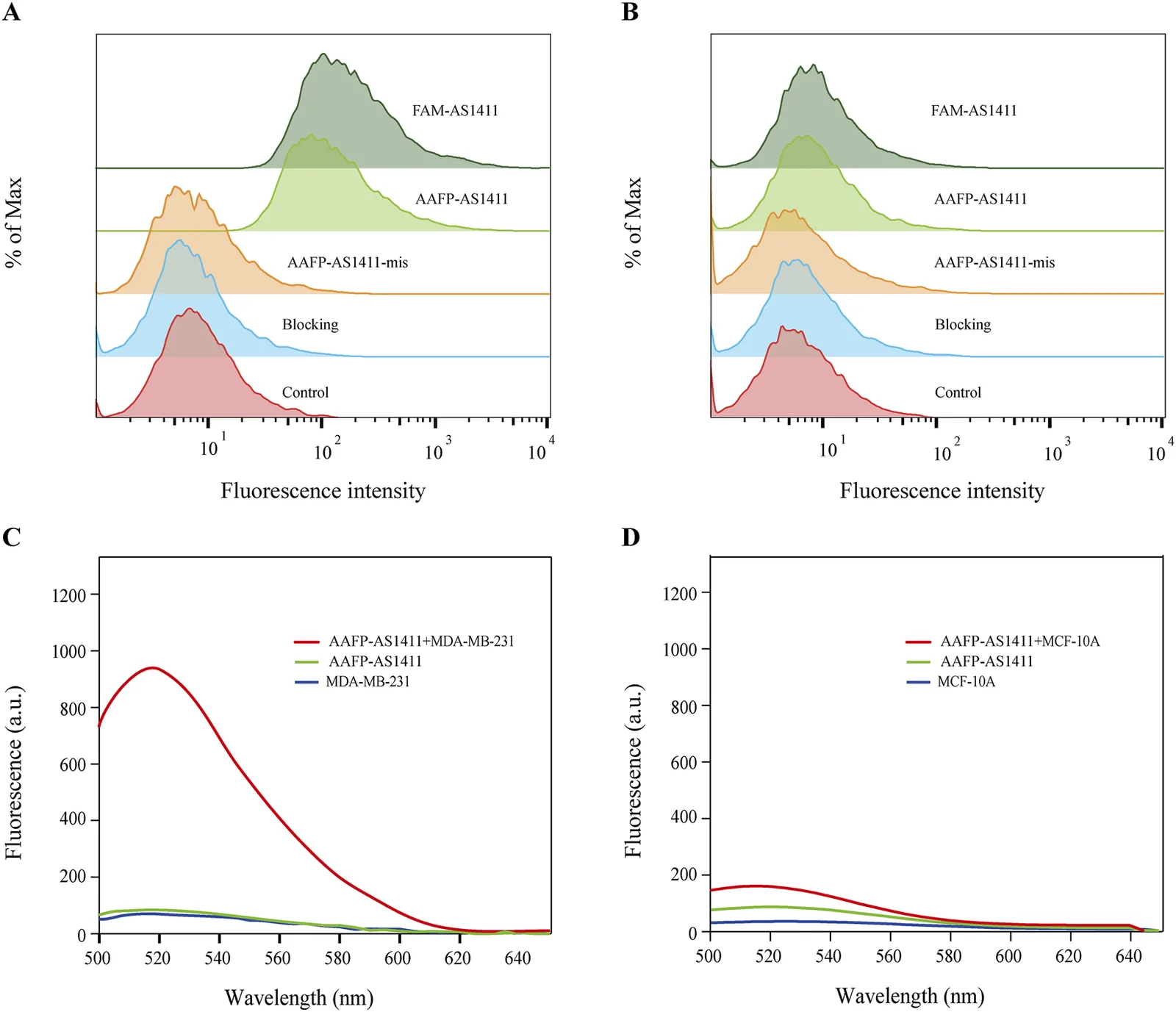
Target binding and fluorescence response of AAFP-AS1411 assessed by flow cytometry and fluorescence spectroscopy. MDA-MB-231 triple-negative breast cancer cells **(A)** and MCF-10A mammary epithelial cells **(B)** were incubated with FAM-AS1411, AAFP-AS1411, the mismatch control AAFP-AS1411-mis, or AAFP-AS1411 following competitive blocking with excess unlabeled AS1411 (Blocking), and subsequently analyzed by flow cytometry. Fluorescence emission spectra were recorded for AAFP-AS1411 in PBS, cells alone (approximately 5 × 10^5^ cells/mL), and mixtures of AAFP-AS1411 with MDA-MB-231 cells **(C)** or MCF-10A cells **(D)** after incubation under the indicated experimental conditions. Flow-cytometry histograms in panels A and B were independently normalized to the percentage of the maximum event count (% of Max) and vertically offset for clarity; fluorescence differences were assessed based on shifts along the fluorescence-intensity axis.

Competitive blocking experiments provided additional evidence for AS1411-associated selectivity. Pre-incubation of MDA-MB-231 cells with excess unlabeled AS1411 substantially reduced the fluorescence signal, consistent with competition for AS1411-associated binding sites.

Fluorescence emission spectra further supported these observations. A strong emission peak at approximately 518 nm was observed when AAFP-AS1411 was incubated with MDA-MB-231 TNBC cells, whereas only minimal emission was detected in MCF-10A cells (Figures 2C,D). Together, these data support a preferential, AS1411-associated fluorescence response in MDA-MB-231 cells under the tested conditions. The reduced fluorescence observed with the mismatch probe and following competitive blocking further supports the selectivity of the probe within this experimental model.

### Optimization of probe conditions

We systematically optimized several parameters governing AAFP-AS1411 performance. First, the length of the complementary C-strand was varied to balance quenching efficiency and activation magnitude. As shown in Figure 3A, the signal-to-background ratio varied with C-strand length, with the 4-base extension providing the highest response among the tested designs. The fluorescence response did not increase monotonically with increasing extension length. The superior performance of the 4-base construct may reflect a more favorable balance between effective fluorescence quenching in the OFF state and sufficient structural flexibility for target-associated fluorescence recovery. In contrast, the 5-base extension may form a relatively more stable complementary interaction, which could partially restrict structural rearrangement and fluorescence recovery upon target engagement. Because the thermodynamic stability and structural rearrangement of these constructs were not directly measured, this interpretation remains hypothetical.

**FIGURE 3:**
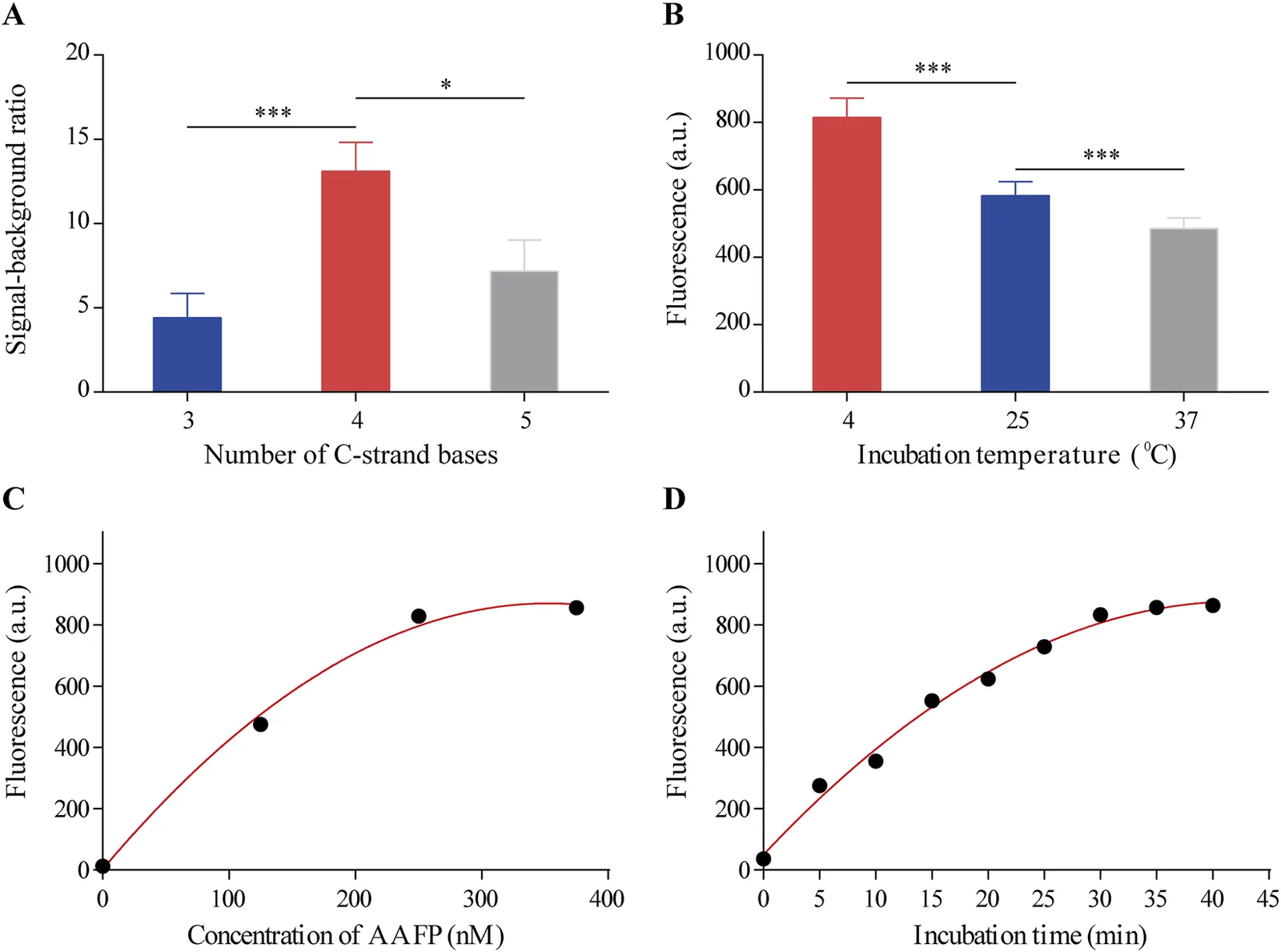
Optimization of C-strand design and assay conditions for AAFP-AS1411. **(A)** Signal-to-background ratios as a function of C-strand length (number of complementary bases). **(B)** Fluorescence of AAFP-AS1411 (250 nM, 30 min) after incubation with MDA-MB-231 cells at 4, 25, or 37 °C. **(C)** Fluorescence response at the indicated AAFP-AS1411 concentrations after 30 min incubation with MDA-MB-231 cells at 4 °C. **(D)** Time-course of fluorescence for AAFP-AS1411 (250 nM) during incubation with MDA-MB-231 cells at 4 °C. Data are presented as mean ± SD (n = 3).

Temperature-dependent fluorescence responses were evaluated by incubating MDA-MB-231 cells with 250 nM probe at 4 °C, 25 °C, and 37 °C for 30 min. As shown in Figure 3B, the strongest fluorescence response was observed at 4 °C. Importantly, a detectable fluorescence response was retained at 37 °C, indicating that probe-associated fluorescence activation was not restricted to the low-temperature condition.

Probe concentration was subsequently evaluated over the range of 0–375 nM (Figure 3C). Fluorescence intensity increased with increasing probe concentration and approached a plateau at approximately 250 nM. Time-course analysis (Figure 3D) showed that the fluorescence response increased progressively during the first 30 min and subsequently plateaued. Based on these observations, 250 nM probe and a 30-min incubation at 4 °C were selected as standardized conditions for the subsequent *in vitro* experiments.

### Concentration-dependent fluorescence response

The fluorescence response of AAFP-AS1411 was evaluated using MDA-MB-231 cells across a range of cell concentrations. Flow cytometry histograms showed concentration-dependent changes in fluorescence (Figure 4A), and the corresponding MFI increased with increasing log_10_-transformed cell concentration (Figure 4B). Consistent concentration-dependent responses were also observed by fluorescence spectroscopy (Figures 4C,D). Regression analysis of the non-zero cell concentrations yielded the relationship *fluorescence = 214.4 × log*_*10*_ *C – 6.981*. The regression was used to describe the concentration–response relationship and was not intended to establish a formal analytical limit of detection.

**FIGURE 4:**
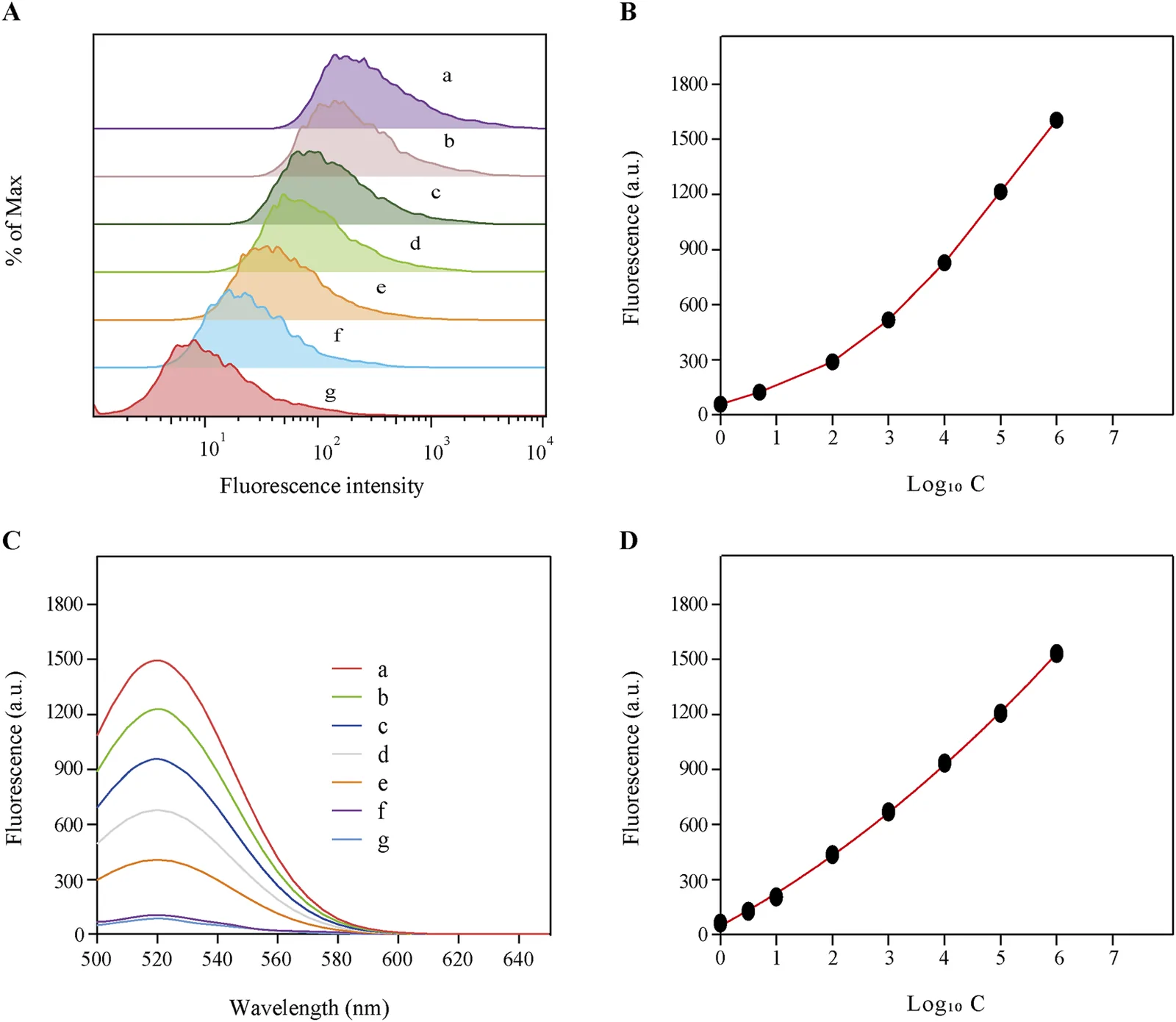
Concentration-dependent fluorescence response of AAFP-AS1411 to MDA-MB-231 cells. **(A)** Flow cytometric analysis of AAFP-AS1411 after 30 min incubation with MDA-MB-231 cells at the indicated concentrations (cells/mL): a, 1 × 10^6^; b, 1 × 10^5^; c, 1 × 10^4^; d, 1 × 10^3^; e, 1 × 10^2^; f, 5; and g, 0. Histograms were independently normalized to % of Max and vertically offset for clarity; the concentration-dependent fluorescence response was evaluated from the horizontal shifts in fluorescence intensity. **(B)** Corresponding mean fluorescence intensity (MFI) obtained by flow cytometry, plotted against log_10_ **(C)**. C represents the cell concentration (cells/mL). Fluorescence emission spectra of AAFP-AS1411 at the indicated cell concentrations: a, 1 × 10^6^; b, 1 × 10^5^; c, 1 × 10^4^; d, 1 × 10^3^; e, 1 × 10^2^; f, 5; and g, 0 (light-blue line). **(D)** Corresponding fluorescence intensity obtained by fluorescence spectroscopy, plotted against log_10_ **(C)**. C represents the cell concentration (cells/mL).

### Fluorescence microscopy and imaging of triple-negative breast cancer cells

Cell-associated fluorescence was further examined by confocal microscopy. MDA-MB-231 cells incubated with FAM-AS1411 or AAFP-AS1411 showed substantially stronger fluorescence than MCF-10A cells (Figures 5A,B). These differences were consistent with the preferential fluorescence response observed by flow cytometry and fluorescence spectroscopy. In contrast, the mismatch probe (AAFP-AS1411-mis) produced only weak fluorescence, indicating that preservation of the AS1411 recognition sequence was important for the observed fluorescence response. Pre-blocking with excess unlabeled AS1411 markedly reduced the fluorescence signal in MDA-MB-231 cells, consistent with competition for AS1411-associated binding sites. Together, these complementary controls support the contribution of sequence integrity and AS1411-associated target engagement to fluorescence activation in the tested cellular model, although they do not directly establish the underlying molecular activation mechanism.

**FIGURE 5:**
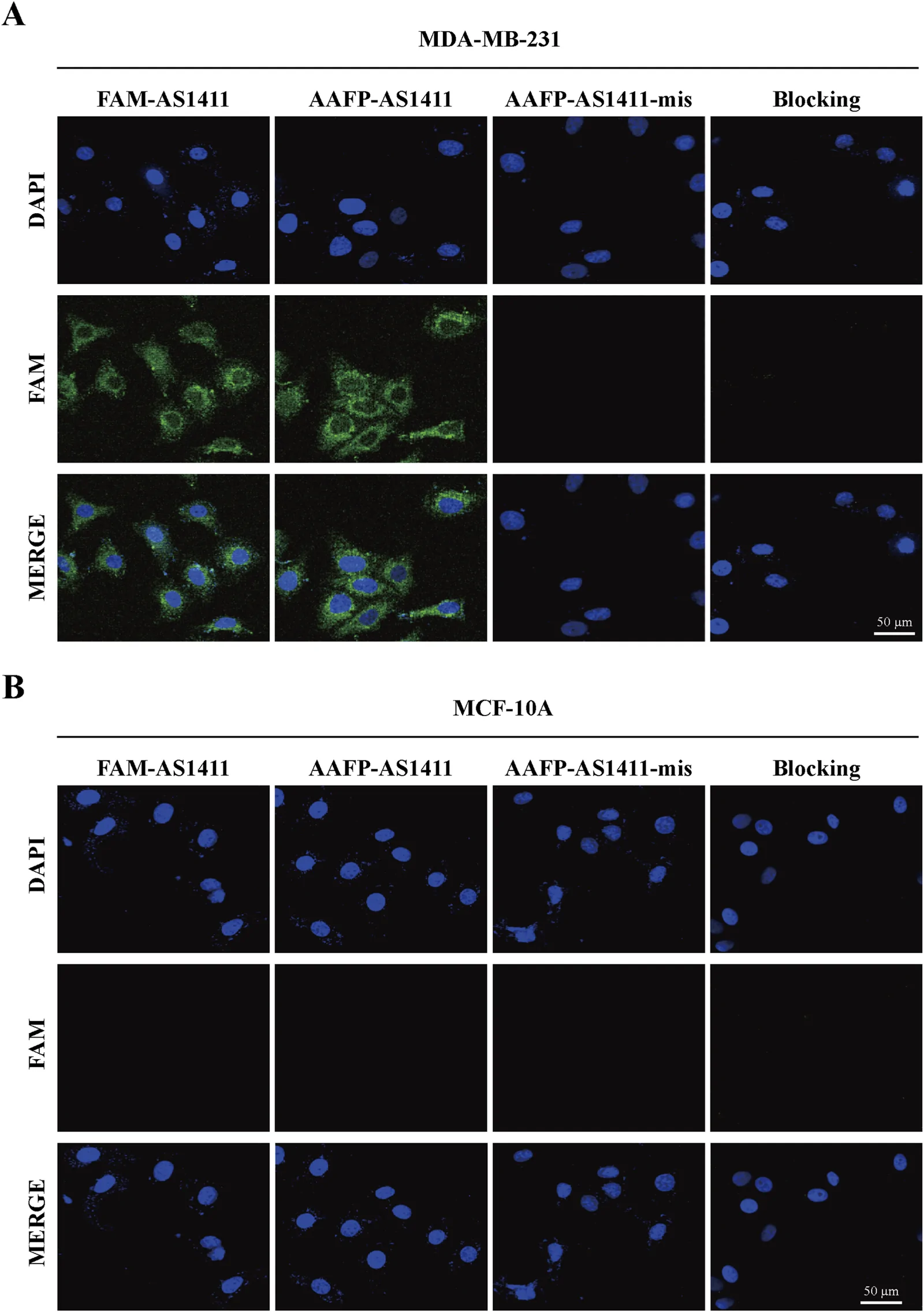
Fluorescence microscopy of probe binding in triple-negative breast cancer and normal mammary epithelial cells. **(A)** MDA-MB-231 and **(B)** MCF-10A cells were incubated with FAM-AS1411, AAFP-AS1411, AAFP-AS1411-mis, or AAFP-AS1411 following competitive blocking with excess unlabeled AS1411 (Blocking). Probe fluorescence was imaged in the green channel, and nuclei were counterstained with DAPI (blue). Scale bar, 50 µm.

### *In Vivo* and *ex vivo* imaging of Cy5.5-AAFP-AS1411

To evaluate tumor-associated fluorescence and its temporal distribution *in vivo*, MDA-MB-231 tumor-bearing BALB/c nude mice were imaged at 1, 6, and 12 h after intravenous administration of Cy5.5-AAFP-AS1411. As shown in Figure 6A, prominent tumor-associated fluorescence was observed in the AAFP-AS1411 group, with the strongest signal detected at 6 h after injection among the examined time points. In comparison, substantially weaker tumor fluorescence was observed in the mismatch and blocking groups. These findings support preferential AS1411-associated fluorescence within the MDA-MB-231 xenograft model.

**FIGURE 6:**
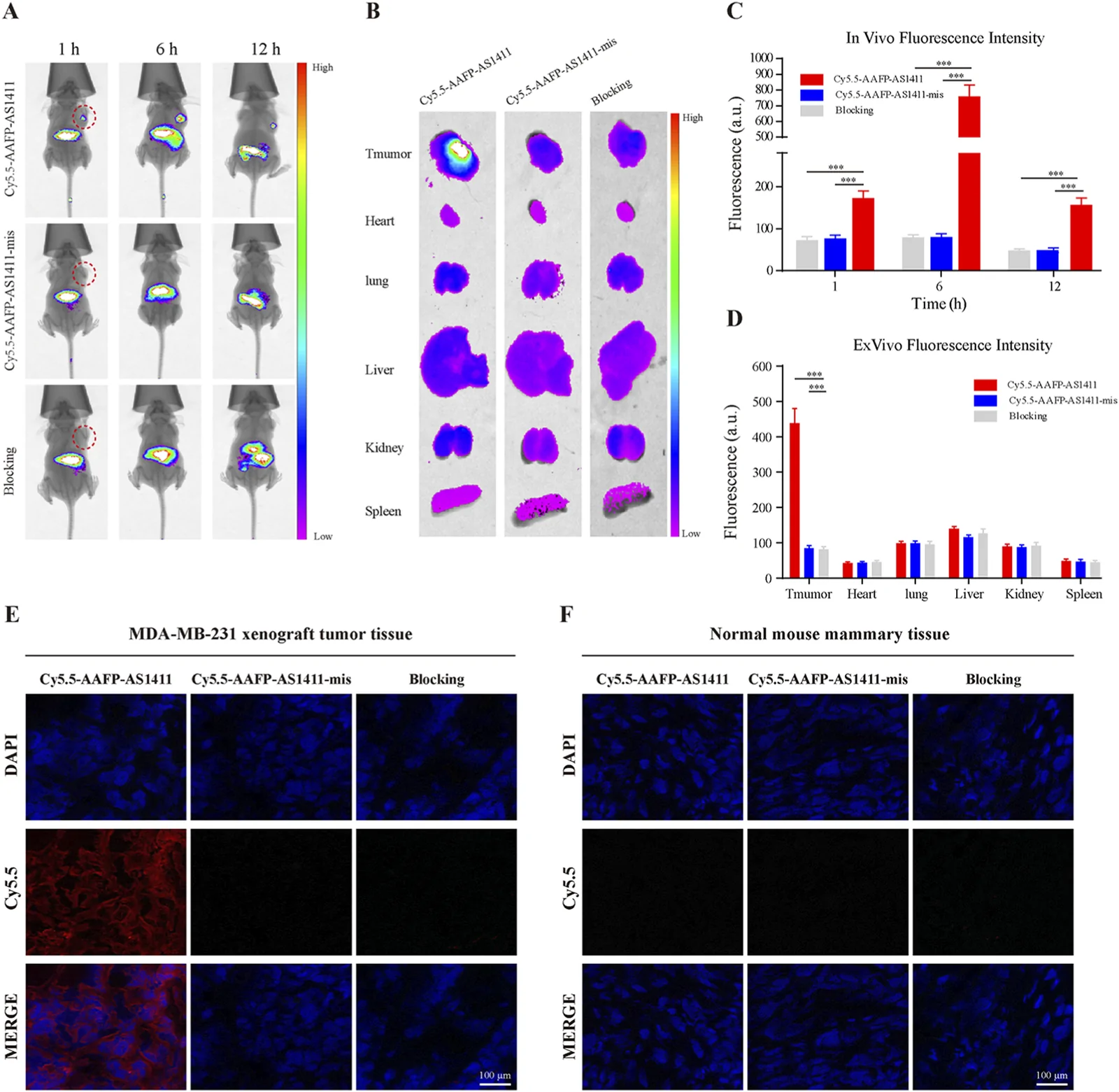
*In vivo* and *ex vivo* fluorescence imaging of MDA-MB-231 tumor-bearing mice. Mice received Cy5.5-AAFP-AS1411, Cy5.5-AAFP-AS1411-mis or Cy5.5-AAFP-AS1411 after pre-injection of excess unlabeled AS1411 (Blocking). **(A)** Time-course *in vivo* fluorescence images of tumor-bearing mice at the indicated time points. Tumor regions are indicated by red circles. **(B)** *Ex vivo* fluorescence imaging of tumors and major organs at 12 h post-injection. **(C)** Quantification of tumor-associated fluorescence at the indicated time points. **(D)** *Ex vivo* ROI-based fluorescence distribution in tumors and major organs at 12 h post-injection. **(E)** *Ex vivo* confocal imaging of tumor tissue. **(F)** *Ex vivo* confocal imaging of contralateral normal mammary tissue. Cy5.5 fluorescence was imaged in the red channel, and nuclei were counterstained with DAPI (blue). Scale bar, 100 µm.

*Ex vivo* fluorescence imaging of tumors and major organs at 12 h further characterized the tissue fluorescence distribution (Figure 6B). The tumor exhibited the strongest fluorescence signal among the examined tissues in mice receiving Cy5.5-AAFP-AS1411, whereas fluorescence signals in the heart, lung, liver, kidney, and spleen were comparatively lower. Tumor fluorescence was also substantially weaker in the mismatch and blocking groups, consistent with an AS1411-associated contribution to the observed tumor signal.

Quantitative ROI analysis (Figure 6C) showed significantly higher tumor fluorescence in the Cy5.5-AAFP-AS1411 group than in the mismatch and blocking groups, with the highest signal observed at 6 h. *Ex vivo* ROI analysis at 12 h (Figure 6D) similarly demonstrated higher fluorescence in tumor tissue than in the examined major organs. Together, these results support preferential tumor-associated fluorescence of Cy5.5-AAFP-AS1411 within the tested xenograft model.

*Ex vivo* confocal microscopy was further performed to compare the fluorescence response of tumor and normal mammary tissues (Figures 6E,F). Tumor sections incubated with Cy5.5-AAFP-AS1411 exhibited substantially stronger red fluorescence than normal mammary tissue sections under the same experimental conditions. These findings provide additional *ex vivo* evidence of preferential probe-associated fluorescence in MDA-MB-231 tumor tissue compared with normal mammary tissue. However, these experiments represent *ex vivo* tissue-level evaluation and should not be interpreted as demonstrating probe penetration or retention following systemic administration.

### Safety evaluation of AAFP-AS1411 in tumor-bearing mice

A preliminary acute safety assessment was performed in MDA-MB-231 tumor-bearing mice following a single intravenous administration of Cy5.5-AAFP-AS1411. As shown in Supplementary Figure S1, hematological parameters (WBC, RBC, HGB, and PLT) and liver and kidney function markers (AST, ALT, BUN, and CREA) showed no statistically significant differences between the probe-treated and PBS-treated groups. Representative H&E-stained sections of the heart, lung, liver, spleen, and kidney showed no obvious treatment-related histopathological abnormalities. These findings indicate no evident acute toxicity at the tested dose and 24-h time point, although the relatively small safety cohort and short observation period warrant further dose-ranging and longer-term safety evaluation.

## Discussion

In this study, we developed an activatable fluorescence probe, AAFP-AS1411, for imaging nucleolin-overexpressing breast cancer. The activatable design allows the probe to maintain relatively low fluorescence before target interaction, thereby reducing background signals compared with constitutively fluorescent probes. In the tested cellular model, AAFP-AS1411 generated substantially stronger fluorescence in MDA-MB-231 TNBC cells than in non-tumorigenic MCF-10A mammary epithelial cells, while fluorescence was markedly reduced with the mismatch probe and following competitive blocking with excess unlabeled AS1411. Consistent findings were observed in the xenograft and *ex vivo* tissue experiments. These results support selective probe activation within the experimental models examined; however, they should not be interpreted as establishing universal specificity across breast cancer subtypes or other nucleolin-expressing malignancies.

The use of the AS1411 aptamer to target nucleolin in oncology is well documented, with established roles in tumor homing, diagnostic imaging, and drug delivery (Kozani et al., 2021; Mehrnia et al., 2021; Khoshnood et al., 2025). Many conventional AS1411-based imaging probes use constitutively fluorescent (“always-on”) reporters, in which fluorescence is generated irrespective of target engagement and may therefore contribute to background signals in complex biological environments (Chen et al., 2017; Chauhan et al., 2019). Activatable AS1411-based strategies have also been reported, supporting the feasibility of coupling nucleolin recognition with fluorescence switching (Zhang et al., 2023). In the present study, AAFP-AS1411 incorporates a fluorophore–quencher architecture designed to suppress fluorescence before target interaction. Based on the probe design, nucleolin interaction is proposed to promote a structural rearrangement that increases fluorophore–quencher separation and results in fluorescence recovery. The markedly attenuated fluorescence produced by the sequence-mismatched probe supports dependence of the cellular response on preservation of the AS1411 recognition sequence, whereas the reduction in fluorescence after pre-incubation with excess unlabeled AS1411 is consistent with competition for AS1411-associated binding sites. Together, these complementary controls support an AS1411-associated target-engagement component in the observed fluorescence recovery, although they do not directly demonstrate the proposed fluorophore–quencher structural transition. This design is conceptually consistent with previously reported activatable aptamer, molecular beacon, and hybridization-switched probe strategies (Lai et al., 2017; Tan et al., 2018; Bura et al., 2022). The specific contribution of the present study is therefore not the introduction of activatable AS1411 sensing itself, but the evaluation of a comparatively simple fluorophore–quencher AS1411 platform across cellular, *in vivo*, and *ex vivo* breast cancer imaging models. Importantly, the target-associated fluorescence behavior observed *in vitro* was retained in the MDA-MB-231 xenograft model, where preferential tumor-associated fluorescence was observed relative to the mismatch and blocking controls. These findings provide proof-of-concept evidence that this activatable architecture can maintain target-associated fluorescence behavior *in vivo* and support further investigation of AAFP-AS1411 as a molecular imaging strategy for nucleolin-overexpressing TNBC.

The present findings suggest several potential directions for further translational investigation. At the *in vitro* level, AAFP-AS1411 exhibited a concentration-dependent fluorescence response across the tested MDA-MB-231 cell concentrations. However, because the regression analysis was not accompanied by independent low-concentration validation, a formal analytical detection limit cannot be established from the present data. Further studies using predefined analytical criteria and independently replicated low-concentration samples will therefore be required to rigorously determine the analytical sensitivity of the probe. In *ex vivo* tissue sections, AAFP-AS1411 generated stronger fluorescence in tumor tissue than in normal mammary tissue under the tested conditions, suggesting potential utility as a molecular contrast tool for tissue-level analysis. In the *in vivo* xenograft model, the probe produced time-dependent tumor-associated fluorescence, with the highest signal observed at approximately 6 h after injection and comparatively lower fluorescence in the examined major organs. These findings provide a rationale for further evaluation of AAFP-AS1411 in applications requiring tumor visualization, including future studies of image-guided surgery. If validated in additional models and patient-derived specimens, such molecular imaging information may ultimately contribute to more refined imaging-guided assessment and clinical decision-making in nucleolin-overexpressing TNBC. However, the present study did not evaluate therapeutic efficacy or treatment response, and the potential role of AAFP-AS1411 in treatment guidance will require dedicated future investigation. In addition, the preliminary safety assessment revealed no evident acute toxicity based on hematological parameters, liver and kidney function tests, and histopathological examination of major organs at the tested dose and time point. Nevertheless, comprehensive pharmacokinetic, biodistribution, dose-ranging, and long-term safety studies will be required before clinical translation can be considered. Taken together, these findings support continued preclinical evaluation of AAFP-AS1411 as an activatable molecular imaging platform for nucleolin-overexpressing TNBC.

An additional consideration is the optical limitation of the fluorophores used in the present study. FAM is particularly susceptible to tissue autofluorescence and limited penetration, while Cy5.5, although operating in the NIR-I window, remains affected by tissue scattering and attenuation during deep-tissue tumor imaging (Antaris et al., 2016). In comparison, NIR-II emitters offer improved penetration and tumor-to-background contrast. For example, CH1055-based NIR-II imaging enabled visualization of orthotopic brain tumors through the intact skull (Kurbegovic et al., 2018), while a recent aptamer-conjugated NIR-II probe demonstrated improved resolution and penetration depth for breast cancer imaging and fluorescence-guided resection (Niu et al., 2025). Future incorporation of suitable NIR-II fluorophores may therefore further improve the deep-tissue imaging performance of AAFP-AS1411. Beyond diagnostic imaging, future studies may also explore theranostic applications of AAFP-AS1411 by linking appropriate therapeutic payloads, potentially integrating AS1411-associated tumor imaging with targeted drug delivery. The feasibility, therapeutic efficacy, and safety of such constructs will require dedicated investigation.

For clinical translation of AAFP-AS1411 as an activatable aptamer probe, GMP-compatible synthesis, purification, and reproducible assembly of the probe components will be required. In addition to conventional assessments of oligonucleotide identity and purity, batch-specific quality control should verify labeling efficiency, OFF-state fluorescence quenching, target-responsive fluorescence recovery, structural and serum stability, and batch-to-batch reproducibility of the activation response. Given that aptamer stability and molecular modification can substantially influence blood clearance and tumor uptake, these parameters should be evaluated together with pharmacokinetics and quantitative biodistribution (Yang et al., 2025; Park et al., 2023). Further preclinical development will also require dose-ranging toxicology, immunogenicity, and other regulatory-compliant safety assessments. Importantly, recent first-in-human pharmacokinetic evaluation of an aptamer has demonstrated the value of directly characterizing systemic distribution, metabolism, and biosafety during clinical translation (Ding et al., 2023). These studies will be necessary to determine whether the activatable fluorescence behavior observed in the present experimental models can be maintained reliably under clinically relevant conditions before progression to early-phase human studies.

Several limitations of the current study should be noted. First, although MDA-MB-231 is a widely used TNBC model, validation in a single TNBC cell line and xenograft model does not capture the molecular and biological heterogeneity of TNBC. Further evaluation in additional TNBC cell lines, molecularly diverse TNBC models, and patient-derived specimens will be needed to determine the robustness and translational applicability of AAFP-AS1411 within TNBC. Second, the preliminary safety assessment was limited to a relatively small cohort (n = 4 per group), a single administration, and a 24-h follow-up. Therefore, the present findings should be interpreted as preliminary evidence of acute safety rather than a comprehensive toxicological assessment. Third, although fluorescence activation remained detectable at 37 °C, most *in vitro* characterization was performed at 4 °C, and further physiological-temperature characterization is warranted. Fourth, the proposed fluorophore–quencher structural rearrangement was inferred from the observed fluorescence responses rather than directly demonstrated and requires further biophysical validation. Future studies should therefore expand validation across diverse TNBC models, characterize probe pharmacokinetics and safety, directly investigate the activation mechanism, and explore advanced imaging applications.

## Conclusion

This study developed an activatable AS1411-based fluorescence probe, AAFP-AS1411, that demonstrated preferential fluorescence activation and high-contrast imaging in the tested MDA-MB-231 TNBC model across *in vitro*, *in vivo*, and *ex vivo* experiments. These findings support further evaluation of AAFP-AS1411 as a molecular imaging strategy for nucleolin-overexpressing TNBC. Validation in additional TNBC models and patient-derived specimens will be required to determine the robustness and translational applicability of this approach within TNBC.

## Data Availability

The main data included in this study are available from the corresponding authors upon reasonable request, while adhering to privacy and ethical guidelines.

## Supplementary material

SUPPLEMENTARY FIGURE S1 Preliminary acute safety evaluation of Cy5.5-AAFP-AS1411 in MDA-MB-231 tumor-bearing mice. **(A)** Hematological parameters 24 h after intravenous administration of PBS or Cy5.5-AAFP-AS1411, including white blood cell count (WBC), red blood cell count (RBC), hemoglobin (HGB), and platelet count (PLT). **(B)** Liver and kidney function parameters, including aspartate aminotransferase (AST), alanine aminotransferase (ALT), blood urea nitrogen (BUN), and creatinine (CREA). **(C)** Representative H&E-stained sections of the heart, lung, spleen, liver, and kidney collected 24 h after administration. Data are presented as mean ± SD (n = 4 per group). Comparisons between the two groups were performed using an unpaired two-tailed Student’s *t*-test. NS, not significant.
